# Supplementary material for: Enhanced Humoral Immune Responses against Toxin A and B of Clostridium difficile is Associated with a Milder Disease Manifestation
Source: J Clin Med. 2020 Oct 10;9(10):3241. doi: 10.3390/jcm9103241 (PMC7601293; doi:10.3390/jcm9103241)
Supplement: Supplementary file 1 [file jcm-09-03241-s001.pdf]

Table S1. Demographic and selected clinical characteristics of cases and control of the original study and the sub-sample included in the current study.

Variable	All cases, *n* = 140	Sub-sample of cases, *n* = 50	All controls, *n* = 140	Sub-sample of controls, *n* = 52
Age, years, Mean (SD)	78.8 (15.4)	79.2 (13.7)	81.4 (7.5)	82.7 (7.6)
Sex (Female), *n* (%)	88 (63%)	31 (62%)	86 (61%)	29 (56%)
Visit to emergency department in the previous year (Yes), *n* (%)	36 (31%)	12 (24%)	43 (31%)	20 (39%)
Antibiotic use (Yes), *n* (%)	117 (84%)	37 (74%)	68 (49%)	25 (48%)
Use of proton pump inhibitors (Yes), *n* (%)	98 (70%)	31 (62%)	101 (72%)	37 (71%)
